# 1-Year Outcomes of Angina Management Guided by Invasive Coronary Function Testing (CorMicA)

**DOI:** 10.1016/j.jcin.2019.11.001

**Published:** 2020-01-13

**Authors:** Thomas J. Ford, Bethany Stanley, Novalia Sidik, Richard Good, Paul Rocchiccioli, Margaret McEntegart, Stuart Watkins, Hany Eteiba, Aadil Shaukat, Mitchell Lindsay, Keith Robertson, Stuart Hood, Ross McGeoch, Robert McDade, Eric Yii, Peter McCartney, David Corcoran, Damien Collison, Christopher Rush, Naveed Sattar, Alex McConnachie, Rhian M. Touyz, Keith G. Oldroyd, Colin Berry

**Affiliations:** a West of Scotland Heart and Lung Centre, Golden Jubilee National Hospital, Clydebank, United Kingdom; b British Heart Foundation Glasgow Cardiovascular Research Centre, Institute of Cardiovascular and Medical Sciences, University of Glasgow, Glasgow, United Kingdom; c Gosford Hospital, NSW Health, Gosford, Australia; d Robertson Centre for Biostatistics, Institute of Health and Wellbeing, University of Glasgow, Glasgow, United Kingdom; e University Hospital Hairmyres, East Kilbride, United Kingdom

**Keywords:** coronary physiology, elective coronary angiography, microvascular angina, stable angina pectoris, stratified medicine, vasospastic angina, ACh, acetylcholine, BP, blood pressure, CAD, coronary artery disease, CFR, coronary flow reserve, CI, confidence interval, FFR, fractional flow reserve, IDP, interventional diagnostic procedure, MACE, major adverse cardiac event(s), MVA, microvascular angina, RR, relative risk, SAQ, Seattle Angina Questionnaire, SAQSS, Seattle Angina Questionnaire summary score, VSA, vasospastic angina

## Abstract

**Objectives:**

The aim of this study was to test the hypothesis that invasive coronary function testing at time of angiography could help stratify management of angina patients without obstructive coronary artery disease.

**Background:**

Medical therapy for angina guided by invasive coronary vascular function testing holds promise, but the longer-term effects on quality of life and clinical events are unknown among patients without obstructive disease.

**Methods:**

A total of 151 patients with angina with symptoms and/or signs of ischemia and no obstructive coronary artery disease were randomized to stratified medical therapy guided by an interventional diagnostic procedure versus standard care (control group with blinded interventional diagnostic procedure results). The interventional diagnostic procedure–facilitated diagnosis (microvascular angina, vasospastic angina, both, or neither) was linked to guideline-based management. Pre-specified endpoints included 1-year patient-reported outcome measures (Seattle Angina Questionnaire, quality of life [EQ-5D]) and major adverse cardiac events (all-cause mortality, myocardial infarction, unstable angina hospitalization or revascularization, heart failure hospitalization, and cerebrovascular event) at subsequent follow-up.

**Results:**

Between November 2016 and December 2017, 151 patients with ischemia and no obstructive coronary artery disease were randomized (n = 75 to the intervention group, n = 76 to the control group). At 1 year, overall angina (Seattle Angina Questionnaire summary score) improved in the intervention group by 27% (difference 13.6 units; 95% confidence interval: 7.3 to 19.9; p < 0.001). Quality of life (EQ-5D index) improved in the intervention group relative to the control group (mean difference 0.11 units [18%]; 95% confidence interval: 0.03 to 0.19; p = 0.010). After a median follow-up duration of 19 months (interquartile range: 16 to 22 months), major adverse cardiac events were similar between the groups, occurring in 9 subjects (12%) in the intervention group and 8 (11%) in the control group (p = 0.803).

**Conclusions:**

Stratified medical therapy in patients with ischemia and no obstructive coronary artery disease leads to marked and sustained angina improvement and better quality of life at 1 year following invasive coronary angiography. (Coronary Microvascular Angina [CorMicA]; NCT03193294)

## Central Illustration

**Figure:**
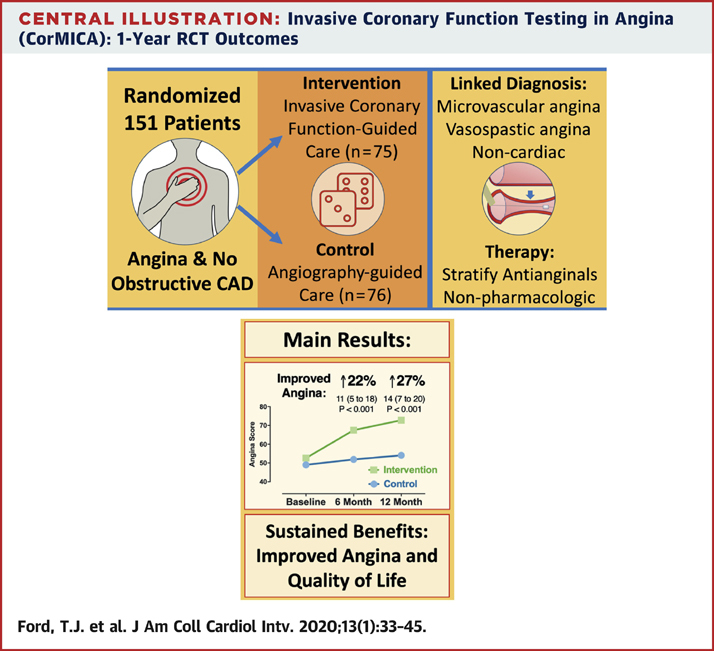

Coronary angiography is routinely performed for the investigation of angina. However, up to one-half of all patients with angina have symptoms and/or signs of ischemia and no obstructive coronary artery disease (CAD) (1). This large, undifferentiated subgroup includes patients with microvascular angina (MVA) and/or vasospastic angina (VSA). These conditions are associated with high morbidity (2), impaired quality of life (3), and considerable use of health resources (4). Furthermore, impaired coronary vasomotion and the propensity to myocardial ischemia may increase longer-term risk for major adverse cardiac events (MACE) (5,6).

In the CorMicA (Coronary Microvascular Angina) trial involving patients with ischemia and no obstructive CAD, we found that an interventional diagnostic procedure (IDP) to rule in or rule out a disorder of coronary vasomotion was feasible and useful. Angina improved more at 6 months in patients whose IDP results were disclosed compared with the blinded control group. We hypothesized that stratified medicine in patients with angina undergoing invasive coronary angiography would benefit patients in the longer term. We thus performed a pre-specified analysis of patient-reported outcome measures at 1 year and assessed longer-term MACE.

## Methods

### Study design

The British Heart Foundation CorMicA trial design and 6-month results have been previously published (7,8). The study is an investigator-initiated, parallel-group, randomized, sham-controlled trial with blinded outcome assessment. We recruited patients with angina without obstructive coronary disease who were randomized immediately after angiography to the intervention (IDP to identify coronary vasomotion disorders with stratified medical therapy of endotypes) or a control group (blinded invasive coronary function testing with standard-care antianginal agents guided by the attending cardiologist).

### Participants

**Figure 1:**
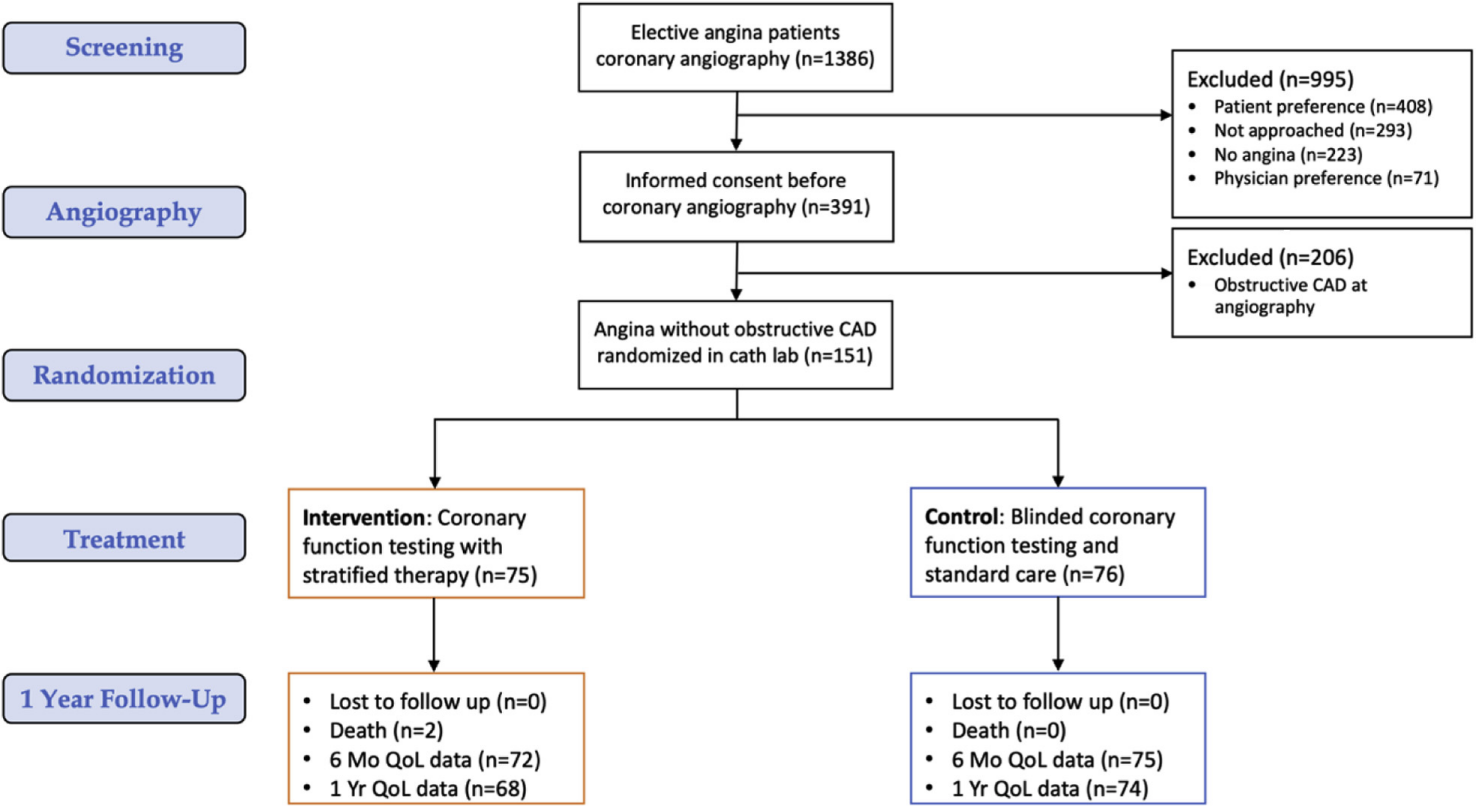
CorMicA Trial Profile According to CONSORT Requirements The total number of patients randomized was 151 with analysis according to intention-to- treat. There was 98% completion of the primary efficacy endpoint assessment at 6 months and 94% at one year.

We screened elective adult referrals to 2 large regional hospitals (Golden Jubilee National Hospital and Hairmyres Hospital) providing invasive cardiac services to all patients in the west of Scotland (population 2.5 million). Outpatients undergoing clinically indicated, elective diagnostic coronary angiography as standard of care for the investigation of angina (definite or probable as defined by the Rose angina questionnaire) were screened and invited to participate (Figure 1) (9). Exclusion criteria were a noncoronary indication for invasive angiography (e.g., valve disease) and inability to give informed consent. Following the provision of informed consent, participants were enrolled on the cardiology ward prior to angiography. Demonstration of obstructive CAD (≥50% diameter stenosis and/or fractional flow reserve [FFR] ≤0.80) during coronary angiography was an exclusion criterion, but these patients entered a registry for ancillary studies. The West of Scotland Research Ethics Committee approved the study (REC 1 reference 16/WS/0192).

### Randomization, groups, and masking

Eligible patients were randomized immediately following angiography 1:1 to the intervention group (IDP plus medical therapy stratified according to IDP results) or control group (IDP performed but results not disclosed [sham]; standard care medical therapy according to physician preference). In other words, all participants underwent the IDP. The results were disclosed to the attending cardiologist in the intervention group and not disclosed in the control group. In the intervention group, the cardiologist reappraised the initial diagnosis on the basis of coronary angiography and could change the diagnosis with linked therapy decisions. In the control group, management was guided by coronary angiography and all of the other available medical information, but not the IDP results. Written guidance informed by practice guidelines was provided to physicians in both groups allowing treatment on the basis of the physicians’ working diagnoses. This included using results of the IDP if available (Online Table 1) (10).

### Blinding and adherence

Patients in the control group had their IDPs performed in the same way as those in the intervention group, except that the results were not disclosed to the treating cardiologists. Details of the blinding procedure have been described (7,8). The outcome assessors and statisticians were blinded to treatment group allocation.

### IDP

The purpose of the IDP was to identify disorders of coronary vasomotion: MVA, VSA, both, or none. Full details of the guidewire-based IDP assessment during adenosine-induced hyperemia and acetylcholine (ACh) provocation are detailed in the Online Appendix.

### Definitions of endotypes

Diagnosis of a coronary vasomotion disorder (MVA, VSA, both, or none) was linked to consensus guideline-based pharmacological and nonpharmacological management (Online Table 1). A diagnosis of VSA required that 3 conditions be satisfied during ACh testing: 1) clinically significant epicardial vasoconstriction (≥90%); 2) reproduction of the usual chest pain; and 3) ischemic electrocardiographic changes (11). MVA was defined according to standardized Coronary Vasomotion Disorders International Study Group diagnostic criteria: symptoms of myocardial ischemia, unobstructed coronary arteries, and proven coronary microvascular dysfunction (any of abnormal index of microcirculatory resistance, coronary flow reserve [CFR], or microvascular spasm to ACh; web appendix on definitions) (9). Diagnosis of coronary microvascular spasm required provocation and reproduction of anginal symptoms, ischemic electrocardiographic shifts, but no epicardial spasm during ACh testing (11). A diagnosis of noncardiac chest pain required no obstructive epicardial CAD (FFR >0.80) and an absence of evidence of any coronary vasomotion disorder (CFR ≥2.0, index of microcirculatory resistance <25, and negative results on ACh testing).

### Stratified medical therapy

After randomization and completion of the diagnostic intervention, research staff members invited the cardiologists to consider the new findings and re-evaluate the diagnosis and treatment plan initially made on the basis of angiography alone. The attending cardiologists in both of the groups were provided with written management guidance specific for each endotype and informed by practice guidelines to facilitate personalized treatment that was specifically aligned to their final diagnosis (Online Appendix) (10). For example, the first-line therapy for MVA incorporates beta-blockers, and nitrates were not recommended, whereas calcium-channel blockers and consideration of long-acting nitrates were advocated for VSA. Standardized letters specific for each endotype were sent to the community-based general practitioners with advice on tailoring and optimizing treatment (including nonpharmacological and lifestyle measures) in line with the final diagnosis (Online Appendix). Standard care for patients in the control group consisted of guideline-directed medical therapy and antianginal therapies according to the preference of the attending cardiologist. To mitigate bias, contacts with participants were standardized between the groups, and all of the participants were managed by the point-of-care clinicians and not the research team.

### Patient-reported outcome measures and health-related quality of life

The Seattle Angina Questionnaire (SAQ) is a self-administered, disease-specific measure of angina severity that is valid, reproducible, and sensitive to change (12). The SAQ summary score (SAQSS) averages the domains of angina limitation, frequency, and quality of life to provide an overall metric of angina severity (13). Full details about patient-reported outcome measure assessments are available in the Online Appendix.

All of the randomized participants were invited to attend for a 1-year follow-up visit in person. The same questionnaire set was completed before the visit. The 1-year visit included 2 additional validated questionnaires to gain insights into exercise and functional capacity. Height, weight, resting pulse rate, and blood pressure (BP) were measured. BP was measured after 5 min of rest in the seated position using a validated oscillometric automated office BP device. If patients declined or were unable to attend at 12 months, the questionnaires were sent with a pre-paid return envelope. Follow-up questionnaires were verified and scored by a blinded member of the research team.

### Clinical events

An independent clinical endpoints committee adjudicated MACE in line with the pre-defined charter. MACE were defined as all-cause death, stroke or transient ischemic attack, unstable angina requiring hospitalization, heart failure requiring hospitalization, or nonfatal myocardial infarction. The definitions for each event are listed in the clinical endpoints committee charter (Online Appendix). Follow-up assessments for serious adverse events were performed up to January 2019. The assessments were performed using electronic National Health Service, a nationwide electronic portal, and sourced individual patient case notes as appropriate.

### 1-year outcomes

#### Primary efficacy endpoint

The primary outcome of this pre-specified analysis was angina severity according to the SAQSS at 1 year (13).

#### Secondary efficacy endpoints

Secondary efficacy endpoints were: 1) health status (including quality of life); 2) lifestyle factors (smoking, weight, BP, and cardiac rehabilitation attendance); 3) physical activity and functional capacity; and 4) MACE.

### Statistical methods

The study design and sample size calculation have been previously described (7).

Health status change from baseline in other domains was analyzed per the primary outcome incorporating baseline score and 6-month score in a mixed-effects linear regression model. The methods are described in the Online Appendix. We performed 2-tailed analyses and considered a p value ≤0.05 to indicate statistical significance. Statistical analyses were performed using R version 3.4.1 (R Foundation for Statistical Computing, Vienna, Austria).

## Results

Between November 2016 and December 2017, we enrolled 391 of 1,386 screened patients (28%) who had been electively referred for invasive coronary angiography with suspected angina (Figure 1). The baseline characteristics of the participants are described in Table 1.

**Table 1:** Baseline Characteristics

	Randomized		
	All Patients (N = 151)	Control (n = 76)	Intervention (n = 75)
Age, yrs	61.0 (53.0–68.0)	60.0 (53.0–68.0)	62.0 (53.5–69.0)
Female	111 (73.5)	58 (76.3)	53 (70.7)
BMI, kg/m^2^	29.7 (25.6–34.7)	29.7 (25.6–34.0)	29.6 (25.7–34.8)
Current smoker	27 (17.9)	14 (18.4)	13 (17.3)
Previous myocardial infarction	24 (15.9)	13 (17.1)	11 (14.7)
Previous stroke or TIA	20 (13.2)	13 (17.1)	7 (9.3)
Diabetes mellitus	29 (19.2)	15 (19.7)	14 (18.7)
Dyslipidemia	120 (79.5)	61 (80.3)	59 (78.7)
Family history of CVD	105 (69.5)	51 (67.1)	54 (72.0)
Predicted 10-yr CHD risk∗	18.6 (10.6–31.4)	18.1 (9.7–27.9)	19.0 (11.9–38.9)
Aspirin	131 (86.8)	67 (88.2)	64 (85.3)
Beta-blocker	101 (66.9)	51 (67.1)	50 (66.7)
Calcium-channel blocker	52 (34.4)	28 (36.8)	24 (32.0)
Nitrates	71 (47.0)	38 (50.0)	33 (44.0)
Statin	126 (83.4)	66 (86.8)	60 (80.0)
Nicorandil	26 (17.2)	15 (19.7)	11 (14.7)
ACE inhibitor or angiotensin receptor blocker	68 (45.0)	35 (46.1)	33 (44.0)
Total cholesterol, mmol/l	3.55 ± 0.98	3.57 ± 1.06	3.52 ± 0.90
HDL cholesterol, mmol/l	1.2 ± 0.4	1.2 ± 0.3	1.2 ± 0.4
Baseline angina questionnaire: nonanginal	0	0	0
Definite (typical) angina	97 (64.2)	42 (55.3)	55 (73.3)
Probable (atypical) angina	54 (35.8)	34 (44.7)	20 (26.7)
Seattle Angina Questionnaire			
Angina summary score	50.8 ± 18.1	49.0 ± 17.2	52.6 ± 18.9
Angina limitation	52.1 ± 24.4	52.4 ± 24.3	51.9 ± 24.7
Angina stability	44.7 ± 24.4	41.4 ± 25.3	48.0 ± 23.2
Angina frequency	59.3 ± 23.5	54.9 ± 21.3	63.7 ± 25.0
Angina treatment satisfaction	81.9 ± 19.5	81.9 ± 20.0	81.8 ± 19.1
Angina quality of life	40.9 ± 21.7	39.7 ± 21.7	42.1 ± 21.9
Quality of life (EQ-5D-5L)			
Index score	0.60 ± 0.29	0.58 ± 0.30	0.62 ± 0.28
VAS score	66.3 ± 20.5	67.9 ± 21.1	64.6 ± 19.8
Stress electrocardiography (performed)	95 (62.9)	46 (60.5)	49 (65.3)
Negative (normal)	13 (13.7)	6 (13.0)	7 (14.3)
Inconclusive	37 (39.0)	18 (39.1)	19 (38.8)
Abnormal	45 (47.4)	22 (47.8)	23 (46.9)
Radionuclide myocardial perfusion (performed)	58 (38.4)	30 (39.5)	28 (37.3)
Negative or inconclusive	28 (48.3)	17 (56.7)	11 (39.3)
Abnormal	30 (51.7)	13 (43.3)	17 (60.7)

Values are median (interquartile range), n (%), or mean ± SD.

ACE = angiotensin-converting enzyme; BMI = body mass index; CHD = coronary heart disease; CVD = cardiovascular disease; HDL = high-density lipoprotein; TIA = transient ischemic attack; VAS = visual analogue scale.

∗ ASSIGN risk score.

The majority of the participants were women (n = 111 [74%]), and the median age was 61 years. There was a high prevalence of cardiovascular risk factors and preventive medicines, in keeping with an elevated 10-year risk for coronary heart disease events (median 18.6%). Antianginal therapy was commonly prescribed (beta-blockers in 101 [67%], long-acting nitrates in 71 [47%], and calcium-channel blockers in 52 [34%]). At randomization, the majority of subjects had daily or weekly angina (SAQ frequency score ≤60), associated with mild to moderate angina limitation (SAQ limitation mean 52.1 ± 24.4). Prior noninvasive stress test results were abnormal in 47% (45 of 95) and 52% (30 of 58) of patients who had abnormal results on exercise stress electrocardiography and radionuclide myocardial perfusion imaging, respectively (Table 1). The mean exercise duration was 6.3 ± 2.6 min with the standard Bruce treadmill exercise test protocol.

Coronary angiography revealed obstructive CAD in 206 patients (53.7%), and 151 of 181 patients (83%) with no obstructive CAD were randomized (n = 75 to the intervention group, n = 76 to the blinded control group). The left anterior descending coronary artery was the target in 88% (n = 132), the right coronary artery in 12 (8%), and the circumflex coronary artery in 6 (4%). Within the randomized population, 74 participants (49%) underwent FFR assessment of CAD as part of standard care. Thirty-four potentially eligible patients were not randomized for logistical and other reasons (Figure 1). The median FFR was 0.88 (interquartile range: 0.84 to 0.92). The median procedure duration (entry to exit from the cardiac catheterization laboratory) was 60 min. The IDP was designed to be performed over an additional 20 min relative to standard-care diagnostic coronary angiography. The endotypes revealed by the IDP in the randomized population included isolated MVA in 78 (52%), isolated VSA in 25 (17%), mixed (both) in 31 (20%), and noncardiac chest pain in 17 (11%). We previously reported data at 6 months showing clinical utility whereby in the intervention arm, physicians were more inclined to include antianginal therapy (87.8% vs. 48.7%; relative risk [RR]: 1.78; 95% confidence interval [CI]: 1.39 to 2.28; p < 0.001). Additionally, in the intervention arm, physicians were more likely to tailor angina therapy specifically to treat a disorder of coronary artery function (86.5% vs. 30.3%; RR: 2.82; 95% CI: 1.98 to 4.02; p < 0.001).

### Primary efficacy endpoint: Angina at 1 year

**Figure 2:**
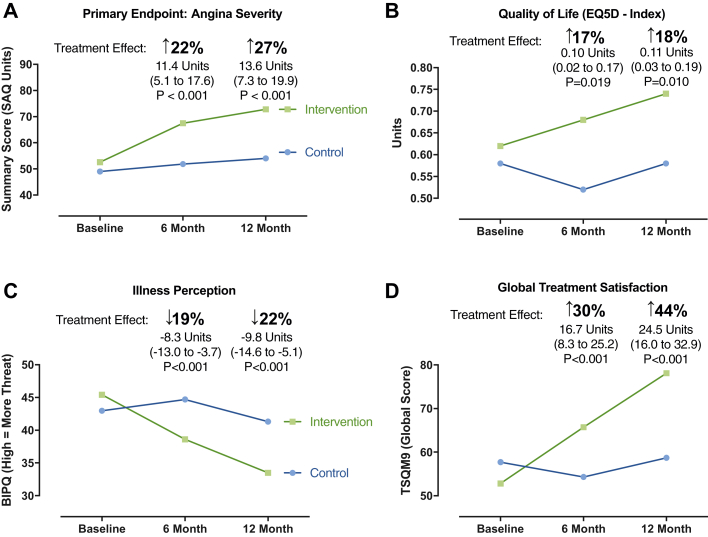
Primary Efficacy Endpoint: Quality of Life Mean Scores at Baseline and at 6 and 12 Months The estimated treatment effect in units is stated with 95% confidence intervals at 6 and 12 months (intervention group and control group). Repeated-measures linear mixed model adjusting for baseline differences between the groups. The relative percentage change represents the estimated treatment effect divided by the mean baseline score for the whole randomized population. **(A)** Primary efficacy endpoint (overall angina severity according to the Seattle Angina Questionnaire [SAQ] summary score). Higher scores represents better (less severe) angina. **(B)** EQ-5D index quality of life (higher scores represent better quality of life). **(C)** Illness perception according to the Brief Illness Perception Questionnaire (BIPQ; higher scores represent a more threatening patient perception of illness). **(D)** Global treatment satisfaction according to the global score of the Treatment Satisfaction Questionnaire for Medication 9 (TSQM-9) validated questionnaire.

One hundred forty-two subjects (94%) completed the 1-year primary outcome assessment (SAQ). No patients were lost to follow-up, and 2 patients (1.3%) died before 1-year follow-up; details of nonresponders are outlined in Figure 1. For the primary endpoint, the SAQSS at 1 year was 27% higher in the intervention group compared with the control group (adjusted mean difference 13.6 units; 95% CI: 7.3 to 19.9; p < 0.001). In practical terms, we observed further separation of angina scores between the 6-month and 1-year time points, representing an incremental difference of 2.3 units (Figure 2). The differences were driven by reduced angina limitation (14.5 units [28%]; 95% CI: 7.9 to 21.1; p < 0.001), reduced angina frequency (9.5 units [16%]; 95% CI: 1.1 to 17.9; p = 0.027), and improved angina-related quality of life (13.6 units [33%]; 95% CI: 7.3 to 19.9; p < 0.001). The individual components of the primary outcome are shown in Table 2 and Figure 2. Subjects with more severe angina (SAQSS below the median) and with more severe psychological distress at baseline had a tendency toward greater improvement in angina with the intervention. There was no interaction between sex, diabetes, or baseline illness perception score with treatment effect.

**Table 2:** Primary Outcome and Changes in Health Status at 1 Year

	Control (n = 76)		Intervention (n = 75)		Treatment Effect at 6 Months			Treatment Effect at 1 Year		
	12 Months	Δ Baseline	12 Months	Δ Baseline	Estimate	95% CI	p Value	Estimate	95% CI	p Value
Primary efficacy endpoint: Seattle Angina Questionnaire										
Summary score	54.2 (24.1)	5.2 (18.0)	72.8 (21.3)	18.4 (21.4)	11.4	5.1 to 17.6	<0.001	13.6	7.3 to 19.9	<0.001
Limitation	51.8 (26.5)	−1.6 (16.2)	67.4 (26.0)	12.4 (21.1)	14.5	8.0 to 21.0	<0.001	14.5	7.9 to 21.1	<0.001
Stability	47.6 (22.0)	6.4 (29.3)	56.3 (22.6)	7.4 (30.9)	4.2	−5.7 to 14.2	0.404	1.9	−8.2 to 12.0	0.716
Frequency	60.8 (27.8)	6.4 (25.3)	80.2 (20.9)	14.7 (27.1)	9.2	0.9 to 17.5	0.030	9.5	1.1 to 17.9	0.027
Satisfaction	80.5 (22.0)	−1.6 (27.9)	94.6 (12.1)	11.7 (18.0)	12.0	5.1 to 19.0	0.001	13.6	6.6 to 20.6	<0.001
SAQ QoL	50.9 (26.1)	11.2 (25.1)	71.9 (24.9)	29.7 (23.6)	11.4	5.1 to 17.6	<0.001	13.6	7.3 to 19.9	<0.001
Secondary efficacy endpoints: health status										
Systolic BP	148.5 (25.3)	15.8 (25.6)	141.8 (22.7)	−1.3 (22.3)	—	—	—	−11.9	−19.3 to −4.5	0.002
Diastolic BP	79.2 (11.0)	8.2 (14.9)	75.9 (11.7)	0.4 (11.7)	—	—	—	−4.8	−8.5 to −1.1	0.011
Weight, kg	83.5 (18.1)	1.2 (4.6)	84.2 (20.3)	−0.2 (11.4)	—	—	—	−1.26	−4.2 to 1.7	0.403
BMI, kg/m^2^	31.0 (6.7)	0.5 (1.9)	30.2 (7.7)	0.0 (4.6)	—	—	—	−0.5	−1.7 to 0.7	0.407
Quality of life (EQ-5D-5L)										
Index score	0.58 (0.34)	−0.01 (0.25)	0.74 (0.24)	0.09 (0.24)	0.10	0.02 to 0.17	0.019	0.11	0.03 to 0.19	0.010
VAS score	67 (22)	−2 (19)	76 (17)	11 (23)	14.5	8.3 to 20.8	<0.001	13.0	6.7 to 19.3	<0.001
Illness perception∗	41 (15)	−2 (17)	34 (14)	−11 (13)	−8.3	−13.0 to −3.7	<0.001	−9.8	−14.6 to −5.1	<0.001
Psychological distress	4.3 (4.3)	−0.5 (3.6)	2.9 (3.6)	−0.7 (3.2)	−0.1	−1.2 to 1.0	0.869	−0.2	−1.3 to 0.9	0.715
Treatment satisfaction										
Effectiveness	65 (21)	6 (24)	77 (22)	20 (27)	11	3 to 19	0.006	13	6 to 21	0.001
Convenience	70 (22)	−4 (22)	86 (16)	18 (20)	14	8 to 21	<0.001	21	14 to 27	<0.001
Global score	59 (27)	0 (26)	78 (21)	26 (27)	17	8 to 25	<0.001	24	16 to 33	<0.001

Treatment effect represents adjusted mean difference at follow-up derived using linear mixed model (intervention − control).

BIPQ = Brief Illness Perception Score; BMI = body mass index; BP = blood pressure; CI = confidence interval; QoL = quality of life; SAQ = Seattle Angina Questionnaire (lower scores represent worse angina symptoms); VAS = visual analogue score of EQ-5D validated quality-of-life tool (higher scores indicate better quality of life).

∗ Illness perception. A higher score reflects a more threatening view of the illness.

### Subgroup analysis

**Figure 3:**
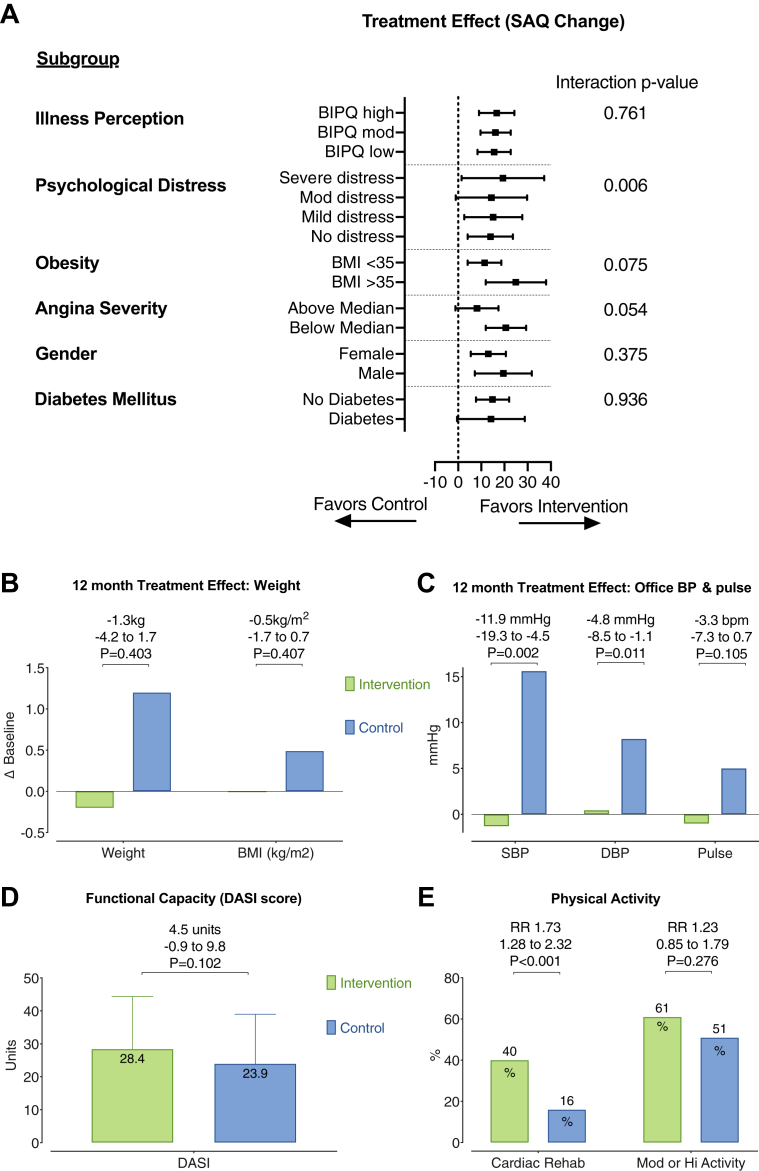
Subgroups and Secondary Endpoints: Weight, BP, and Physical Activity Analyses **(A)** to **(C)** used linear regression adjusting for baseline value. Mean treatment effect is displayed for each groups with its 95% confidence interval and statistical significance. **(A)** Analysis of subgroup interaction with estimated treatment effect. **(B)** Estimated 1-year mean change from baseline in body weight and body mass index (BMI) between groups (intervention, **green**; control, **blue**). **(C)** Estimated 1-year mean change from baseline in systolic blood pressure (SBP), diastolic blood pressure (DBP), and pulse (intervention, **green**; control, **blue**). **(D)** Average functional capacity at 1 year in each group as measured using the Duke Activity Status Index (DASI). **Bars** represent mean score ± SD. Unpaired Student’s *t*-test for significant difference in DASI score between groups. The estimated difference between the groups and its 95% confidence interval are displayed. **(E)** Proportion of subjects in each group participating in cardiac rehabilitation or participating in “moderate” or “high” physical activity according to the International Physical Activity Questionnaire (IPAQ). BIPQ = Brief Illness Perception Questionnaire; BP = blood pressure; RR = relative risk as a measure of effect size with 95% confidence interval and statistical significance for each domain.

The subgroup analysis for the interaction of baseline characteristics and estimated treatment effect is shown in Figure 3. There were clinically relevant between-group differences in prescribed therapies stratified by endotype at 12 months. Patients in the intervention arm with VSA were more likely to be taking calcium-channel antagonists at 12 months compared with those in the control arm, whereas patients with MVA were more likely to be taking beta-blockers and angiotensin-converting enzyme inhibitors at 12 months compared with those in the control arm (Table 3). Interestingly, there was no significant treatment effect in the noncardiac chest pain group (95% CI: −6.9 to 34.2; p = 0.212). Otherwise the MVA group seemed to have the most statistically significant treatment effect (2.0 to 20.1 units; p = 0.019). Nevertheless, a subgroup analysis in this small study is underpowered, while overall estimated average treatment effect between the groups was numerically similar but with wider CIs in the smaller groups.

**Table 3:** Prescribed Therapies According to Randomized Group and Diagnosis Revealed by IDP

	Noncardiac			MVA			VSA			Mixed (MVA and VSA)		
	Intervention (n = 6)	Control (n = 11)	p Value	Intervention (n = 43)	Control (n = 35)	p Value	Intervention (n = 12)	Control (n = 13)	p Value	Intervention (n = 14)	Control (n = 17)	p Value
Aspirin	1 (16.7)	7 (63.6)	0.131	30 (69.8)	19 (54.3)	0.239	11 (91.7)	7 (53.8)	0.073	12 (85.7)	13 (76.5)	0.664
Beta-blocker	1 (16.7)	7 (63.6)	0.131	29 (67.4)	15 (42.9)	**0.039**	2 (16.7)	6 (46.2)	0.202	7 (50.0)	12 (70.6)	0.288
CCB	2 (33.3)	5 (45.5)	1.000	20 (46.5)	10 (28.6)	0.160	7 (58.3)	2 (15.4)	**0.041**	8 (57.1)	3 (17.6)	**0.031**
Nitrates	0 (0.0)	3 (27.3)	0.515	8 (18.6)	13 (37.1)	0.078	9 (75.0)	4 (30.8)	**0.047**	9 (64.3)	5 (29.4)	0.076
Nitroglycerin	4 (66.7)	5 (45.5)	0.620	37 (86.0)	21 (60.0)	**0.018**	10 (83.3)	7 (53.8)	0.202	14 (100.0)	6 (35.3)	**<0.001**
Nicorandil	0 (0.0)	1 (9.1)	1.000	8 (18.6)	6 (17.1)	1.000	4 (33.3)	4 (30.8)	1.000	3 (21.4)	2 (11.8)	0.636
ACE inhibitor or ARB	3 (50.0)	7 (63.6)	0.644	26 (60.5)	11 (31.4)	**0.013**	5 (41.7)	4 (30.8)	0.688	10 (71.4)	7 (41.2)	0.149
Statin	2 (33.3)	8 (72.7)	0.162	37 (86.0)	20 (57.1)	**0.005**	11 (91.7)	8 (61.5)	0.160	13 (92.9)	10 (58.8)	**0.045**
Ranolazine	0 (0.0)	0 (0.0)	1.000	0 (0.0)	2 (5.7)	0.198	0 (0.0)	0 (0.0)	1.000	1 (7.1)	0 (0.0)	0.452
Ivabradine	0 (0.0)	0 (0.0)	1.000	0 (0.0)	1 (2.9)	0.449	1 (8.3)	1 (7.7)	1.000	0 (0.0)	1 (5.9)	1.000

Values are n (%) unless otherwise indicated. Randomized groups were compared using Fisher exact tests without multiplicity correction. **Bold** indicated significance between group differences in therapies at six months.

ACE = angiotensin-converting enzyme; ARB = angiotensin receptor blocker; CCB = calcium-channel blocker; IDP = interventional diagnostic procedure; MVA = microvascular angina; VSA = vasospastic angina.

### Secondary efficacy endpoints

#### Health status (including quality of life)

Patient-reported quality of life at 1 year according to the EQ-5D-5L visual analogue scale was significantly improved in the intervention group (difference 13.0 units [20%]; 95% CI: 6.7 to 19.3; p < 0.001). Similarly, the EQ-5D-5L index also improved in the intervention relative to control group (mean difference 0.11 units [18%]; 95% CI: 0.03 to 0.19; p = 0.010) (Figure 2). Patient global treatment satisfaction was 44% higher in the intervention group (24.5 units; 95% CI: 16.0 to 32.9; p < 0.001; Figure 2). Illness perception scores were significantly lower at 1 year, reflecting a less threatening perception of illness in the intervention group relative to control (−9.8 units [−22%]; 95% CI: −14.6 to −5.1; p < 0.001). There were no between-group differences in psychological distress scores at follow-up (Patient Health Questionnaire 4 treatment effect −0.2 units; 95% CI: −1.3 to 0.90; p = 0.715).

#### Lifestyle factors: weight, BP, cardiac rehabilitation, and smoking

One hundred thirty-three subjects (88%) attended the 1-year study assessment for in-person BP and anthropometric measurements. Systolic and diastolic BPs were both lower in the intervention group at 1-year follow-up (systolic BP −11.9 mm Hg; 95% CI: −19.3 to −4.5 mm Hg; p = 0.002; diastolic BP −4.8 mm Hg; 95% CI: −8.5 to −1.1 mm Hg; p = 0.011). Importantly, this effect was associated with a rise in systolic BP from baseline in the control group (median 13.5 mm Hg), whereas there was only a modest decrease in the systolic BP change from baseline in the intervention group (median −2 mm Hg). Patient participation at cardiac rehabilitation was higher in the intervention group (40% vs. 16%; RR: 2.53; 95% CI: 1.41 to 4.56; p = 0.001). Active smoking at 1 year was similar between the groups (12% vs. 15%; RR: 0.84; 95% CI: 0.37 to 1.91; p = 0.678). The adjusted mean difference in weight from baseline to 1 year was not statistically significant between the intervention (−0.2 kg) and control (1.2 kg) groups (estimated treatment effect −1.26 kg; 95% CI: −4.23 to 1.71; p = 0.403). Body mass index change was not statistically different between the groups (−0.51 kg/m^2^; 95% CI: −1.71 to 0.70 kg/m^2^; p = 0.407).

#### Physical activity and functional capacity

Physical activity assessed using the International Physical Activity Questionnaire–Short Form at 12 months was numerically higher in the intervention group at follow-up, but the differences were not statistically significant different between the groups (total exercise metabolic equivalent minutes per week [intervention vs. control] median 1,386 vs. 1,188; p = 0.072). Categorization into moderate and high physical activity levels was also not different between the groups (60% moderate/high in intervention group vs. 51% in control group; RR: 1.19; 95% CI: 0.88 to 1.61; p = 0.266).

Estimated functional capacity from the Duke Activity Status Index was not different (6.2 ± 2.0 vs. 5.7 ± 1.9; p = 0.102). The overall Duke Activity Status Index score was 4.5 units higher in the intervention group compared with the control group (95% CI: −0.9 to 9.8; p = 0.102), but this result was not statistically significant.

#### MACE

During a median period of 19 months (interquartile range: 16 to 22 months), 9 subjects (12%) in the intervention group and 8 (11%) in the control group experienced MACE (p = 0.803). Overall, 2 participants (1%) died, 4 (3%) experienced nonfatal myocardial infarction, 3 had cerebrovascular events (2%), 2 (1%) were hospitalized for heart failure, and 9 (6%) experienced unstable angina requiring urgent revascularization or hospitalization. Causes of death were cardiac (heart failure, n = 1) and noncardiac (malignancy, n = 1). These events are detailed in Table 4. There were no between-group differences in any of the MACE subtypes during longer term follow-up.

**Table 4:** Secondary Endpoints: Physical Activity, Health Promotion, and Clinical Events

	Control (n = 76)	Intervention (n = 75)	p Value
Physical activity (12 months)			
Physical activity (IPAQ-SF)			
MET minutes per week	1,188 (173–2,532)	1,386 (462–3,861)	0.072
Moderate or high physical activity levels	36 (51)	38 (60)	0.528
Functional capacity (DASI)			
Estimated peak VO_2_	19.9 ± 6.5	21.8 ± 6.9	0.102
Estimated METs	5.7 ± 1.9	6.2 ± 2.0	0.102
Overall DASI score	23.9 ± 15.1	28.4 ± 16.0	0.102
Cardiac rehabilitation	12 (16)	30 (40)	**0.001**
Smoking	11 (15)	9 (12)	0.811
Clinical events (19 months)∗			
MACE	8 (10.5)	9 (12.0)	0.803
Death	0 (0.0)	2 (2.7)	0.245
Myocardial infarction	2 (2.6)	2 (2.7)	1.000
Stroke/TIA	2 (2.6)	1 (1.3)	1.000
Unstable angina (hospitalization or revascularization)	5 (6.6)	4 (5.3)	1.000
Heart failure (hospitalization)	0 (0.0)	2 (2.7)	0.245

Values are median (interquartile range), n (%), or mean ± SD. Randomized groups were compared using Fisher exact test for categorical variables and Student’s *t*-test for continuous variables. The median duration of follow-up was 19 months (range: 16 to 22 months). Causes of death in patients were cardiovascular (heart failure, n = 1) and noncardiovascular (cancer, n = 1).

DASI = Duke Activity Status Index (estimates functional capacity); IPAQ-SF = International Physical Activity Questionnaire–Short Form; MACE = major adverse cardiac events; MET = metabolic equivalent units; TIA = transient ischemic attack; VO_2_ = maximum rate of oxygen consumption measured during incremental exercise; other abbreviations as in Tables 1 and 3.

∗ Mann-Whitney Wilcoxon test. **Bold** indicates p < 0.05.

## Discussion

We found that angina severity, quality of life, treatment satisfaction, and illness perception improved at 1 year in the stratified therapy intervention group relative to control. We observed mechanistic differences that help explain the treatment effect, notably appropriate stratification of therapy, lower systolic and diastolic BPs relative to control, enhanced participation in cardiac rehabilitation, and nonsignificant trends toward improved functional capacity and physical activity levels in the intervention group (Central Illustration). There were no procedural safety concerns, and MACE were appreciable in the randomized population, with no significant between-group differences.

**Central Illustration:**
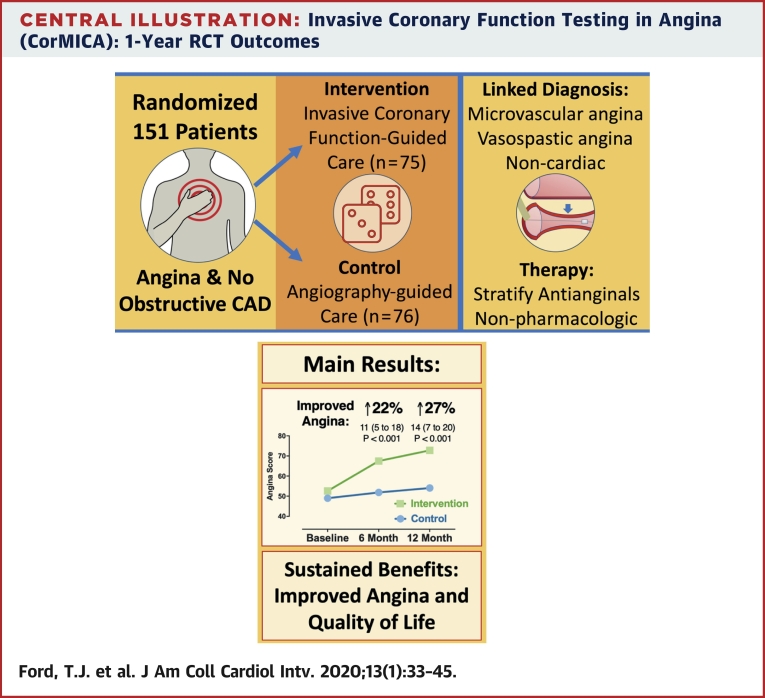
Invasive Coronary Function Testing in Angina (CorMICA): 1-Year RCT Outcomes CAD = coronary artery disease; CorMICA = Coronary Microvascular Angina; RCT = randomized controlled trial.

The 1-year difference in angina severity reflected progressive differences over time associated with stratified therapy. The magnitude of the treatment effect relative to the baseline score (14 units of the SAQSS; 95% CI: 7 to 20 units; p < 0.001) represented a 27% higher overall angina score. This is consistent with 1 grade in the Canadian Cardiovascular Society classification and a clinically meaningful difference for patients (14). This improvement is greater than the minimum clinically important difference of 8 points for the SAQ angina limitation, frequency, and quality-of-life domains and 5 points for SAQ treatment satisfaction (15). The increment from baseline to 1 year in the EQ-5D-5L index was 0.06 units (95% CI: 0.00 to 0.21), and the treatment effect (between-group difference) was 0.11 units (95% CI: 0.03 to 0.19; p = 0.010). The ORBITA trial enrolled patients with obstructive CAD, and patients randomized to percutaneous coronary intervention had a 1-month increment of 0.03 units (95% CI: 0.00 to 0.06), with no between-group difference between percutaneous coronary intervention and sham control (16). CorMicA participants had a much higher burden of health impairment (SAQ and EQ-5D), potentially indicating greater scope for health gain from a personalized intervention including cardiac rehabilitation. We observed a significant interaction between psychological distress at baseline and subsequent treatment response, highlighting the importance of addressing psychological factors that contribute to patients’ experience of angina (17).

### Pharmacological treatment effect

Stratified medicine is the identification of key subgroups of patients (endotypes) within an undifferentiated, heterogeneous population, these endotypes (MVA, VSA, both, or none) being distinguishable by distinct mechanisms of disease and/or responses to therapy (18). We observed between-group differences in medical therapies by endotype at 1 year, indicating personalized therapy. In addition, there were more prescribed antianginal and ischemic heart disease therapies in the intervention group at 1 year (median 4 [interquartile range: 3 to 5] vs. 3 [interquartile range: 1 to 4] in the control group). Improvements in health status may in part relate to higher use of angiotensin-converting enzyme inhibitors and statins, agents with disease-modifying properties with plausible benefits on microcirculatory and endothelial function (19,20). Resting systolic and diastolic BPs were lower at 1 year in the intervention group, an effect that could be mediated by a combination of a larger number of antianginal therapies, better therapy compliance, and less inappropriate cessation of therapy.

### Nonpharmacological treatment effect

Patients with newly diagnosed angina or ischemic heart disease may benefit from cardiac rehabilitation, and we observed more than 2-fold use in the intervention group (40% vs. 16%; RR: 2.53; 95% CI: 1.41 to 4.56; p = 0.001). Cardiac rehabilitation improves functional and physical exercise capacities and may have important psychological benefits, helping patients understand their illness (21). One-half of all participants had body mass index >30 kg/m^2^ at baseline, and we did not observe any significant between-group differences in weight or body mass index at 1-year follow-up. Interestingly, there was a trend toward a higher treatment response in extremely obese patients (body mass index >35 kg/m^2^), which could be the focus of further research. Strategies of intensive weight loss have shown disease-modifying properties in people with diabetes (22).

Angina symptoms are often subjective and multifactorial in origin; listening to patients and providing education and explanation or validation of symptoms may facilitate improvement in angina (23). Indeed, it is impossible to fully separate the impact of a definitive diagnosis on symptoms and the benefits achieved related solely to pharmacological therapy. A conclusive diagnosis may be therapeutic in itself (24). The effect of having a diagnosis may motivate patients to modify lifestyle and possibly improve compliance to a much greater extent than those in the control group, who did not have the benefit of receiving a correct diagnosis. Illness perception may be more threatening in patients with diagnostic uncertainty, and this is an important predictor of longer term disability and not returning to work (25).

### Study limitations

First, we adopted binary cutoffs for the IDP test results in line with guidelines and established diagnostic thresholds. The optimal prognostic thresholds for these parameters of ischemia (e.g., CFR, index of microcirculatory resistance, ACh response) are part of a continuum. It is possible that indeterminate (gray-zone or borderline) test results may be misclassified (26). Nevertheless, we adopted a stringent approach using unambiguous reference thresholds for disease classification (e.g., CFR cutoff of 2.0 rather than 2.5). The IDP was focused on a single major coronary artery for pragmatic reasons to avoid unnecessarily prolonging the procedure. In patients with microvascular disease, regional variations in myocardial blood flow at rest and during pharmacological hyperemia may be detected by quantitative imaging with positron emission tomography and cardiac magnetic resonance (6,27). Importantly, these noninvasive tools have not been validated for diagnosing vasospastic disorders.

Second, we performed ACh provocation testing after the administration of glyceryl trinitrate for assessment of coronary function during adenosine-induced hyperemia. There is no firm consensus on the timing of whether ACh testing should be before or after adenosine testing. We advocate ACh testing after adenosine because a markedly positive result for vasospasm may confound the assessment of resting blood flow because of elevated sympathetic drive (i.e., CFR may be falsely lowered). In contrast, the half-life of glyceryl trinitrate is about 2 min (28), and a false-negative result for coronary vasospasm is thus unlikely following the first stage of the IDP (adenosine).

Finally, a simple and pragmatic approach would be to treat all patients with possible angina and nonobstructive CAD with an additional antianginal therapy as a therapeutic trial. As clinical researchers, we believe that a person-centered approach is paramount. Optimizing therapy to a specific diagnosis and avoiding harm from unnecessary long-term polypharmacy will benefit patients and health care providers. Furthermore, stratifying this undifferentiated patient cohort paves the way for developing disease-modifying therapy. In this regard, we have shown that endothelial dysfunction and endothelin-1 dysregulation are important and may represent potential therapeutic targets for patients with symptoms and/or signs of ischemia and no obstructive CAD (29). CorMicA highlights the limitations of anatomic tests for identifying coronary vasomotion disorders. Indeed, anatomic testing (e.g., computed tomographic coronary angiography) may result in false reassurance for patients with no obstructive CAD but underlying MVA and/or VSA. These patients are predominantly women (30). Discontinuation of therapy by protocol in patients with undiagnosed MVA may be one explanation for why management guided by computed tomographic coronary angiography is associated with more angina and worse health-related quality of life compared with standard care (31).

## Conclusions

Invasive coronary physiological assessment allows stratified medical therapy, representing an opportunity for better long-term angina treatment in patients without obstructive CAD. Larger multicenter trials and cost-effectiveness analyses are needed.

Perspectives **WHAT IS KNOWN?** In patients with angina, stratified medicine improves angina and quality of life in the short term. Whether these improvements are sustained in the longer term is unknown.

**WHAT IS NEW?** Invasive coronary physiology can help to identify distinct treatable subgroups within the angina population without obstructive CAD. Stratified medicine led to sustained improvements in angina and well-being. Mechanisms included diagnostic reclassification with linked therapy including cardiac rehabilitation.

**WHAT IS NEXT?** More trials are needed to extend external validity and expand the evidence base.
