# Supplementary material for: 1-Year Outcomes of Angina Management Guided by Invasive Coronary Function Testing (CorMicA)
Source: JACC Cardiovasc Interv. 2020 Jan 13;13(1):33–45. doi: 10.1016/j.jcin.2019.11.001 (PMC8310942; doi:10.1016/j.jcin.2019.11.001)
Supplement: Online Appendix and Online Table 1 [file mmc1.docx]

Supplementary Appendix

This appendix has been provided by the authors to give readers additional information about the CorMicA one-year outcomes paper.

Table of Contents

[1. Supplementary Methods 3](#_Toc23927676)

[Interventional Diagnostic Procedure (IDP) 3](#_Toc23927677)

[Statistical Considerations 3](#_Toc23927678)

[Patient reported outcome measures (PROMS) and health-related quality of life 5](#_Toc23927679)

[2. Table 1 - Diagnosis and stratified treatment of coronary vasomotion disorders. 7](#_Toc23927680)

[3. Table 2 - Patient demographics at baseline according to INOCA endotype 9](#_Toc23927681)

# Supplementary Methods

## Interventional Diagnostic Procedure (IDP)

The standard care invasive coronary angiogram was performed via the radial artery wherever feasible. Coronary function tests were performed as an adjunctive procedure to the angiogram after randomization in the catheter laboratory. The IDP was focused on a single major coronary artery for pragmatic reasons to avoid unnecessarily prolonging the procedure. The left anterior descending coronary artery was the prespecified target vessel however if technical factors precluded guidewire-based assessment of this artery e.g. tortuous anatomy, then the left circumflex or right coronary artery was selected. Full details of the diagnostic procedure are provided in the appendix. In brief, the IDP involved passing a diagnostic coronary guidewire via a guiding catheter for assessment of coronary flow reserve (CFR; abnormal <2.0), the index of microcirculatory resistance (IMR; abnormal ≥25) and fractional flow reserve (FFR, abnormal ≤0.80) during intravenous infusion of adenosine (140 µg/kg/min). Incremental concentrations of acetylcholine (ACh) (10^-6^M, 10^-5^M, 10^-4^M) were then sequentially infused during 2-minute periods, followed by vasospasm provocation testing (ACh bolus, 100 μg for left coronary artery or 50 μg right) and finally 300 µg of glyceryl trinitrate (GTN).

## Statistical Considerations

### Role of the funding source

The funder (British Heart Foundation) had no role in the study design, data collection, analysis, interpretation, or writing of the report. The corresponding author had full access to all the data in the study and had final responsibility for the decision to submit for publication.

### Randomization procedure

Trained staff in the catheter laboratory used a web-based randomization tool to immediately randomize the patient after the index coronary angiography revealed no obstructive CAD (Robertson Centre for Biostatistics , University of Glasgow). The randomization sequence involved block lengths of 4 and 6, randomized in blocks of 4, that is, every 20 allocations consists of 4 blocks, 2 of length 4 and 2 of length 6, in a random order. Patients were immediately randomized using a web-based portal.

### Statistical Analysis

The study design and sample size calculation for the six month primary outcome of the CorMicA study has been previously published.(10) Continuous outcome measures recorded at baseline, six months and one year were compared between randomized groups using a mixed effects linear regression model, including a random effect for patients, and fixed effects for time point (baseline or follow-up), randomized group, and their interaction. The baseline-adjusted treatment effect was estimated as the interaction term from this model. To assist with interpretation of effect size, the adjusted mean change from baseline was expressed as a relative percentage change using the estimated treatment effect divided by the mean baseline score for the whole randomized population.(22)

Health status change from baseline in other domains was analyzed as per the primary outcome incorporating baseline score and six month score in a regression model. All available patient data was included including subjects with incomplete follow up data. These were considered to be missing at random.

The study population comprised all of the participants who had provided informed consent and no patients were excluded. There were no interim analyses and the trial enrolment was considered complete after the prespecified recruitment target was met. Data are reported as mean (standard deviation, SD), median (25^th^, 75^th^ percentile), or frequency and percentage. Categorical outcomes were compared between randomized groups using Fisher's Exact Tests with additional calculation of effect size (as relative risk). We performed 2-tailed analyses and considered a P value ≤ 0·05 to be significant. Statistical analyses were performed using R v3.4.1.

## Patient reported outcome measures (PROMS) and health-related quality of life

The SAQ quantifies patients’ physical limitation caused by angina, the frequency of and recent changes in their symptoms, their satisfaction with treatment, and the degree to which they perceive their disease to affect their quality of life. Each scale is transformed to a score of 0 to 100, where higher scores indicate better function (eg, less physical limitation, less angina, and better quality of life).(10) Health status was serially assessed using validated, self-administered questionnaires for quality of life using the EuroQOL (EQ5D-5L). This is a widely used standardized instrument for measuring generic health status whereby higher scores represent better quality of life ( from −0.59 – 1.00 scale).(12) We recorded the illness perception using the validated brief illness perception questionnaire (B-IPQ),(13) anxiety and depression according to the PHQ–4(14) and treatment satisfaction (TSQM-9)(15). At six months, the patients’ anginal symptoms were assessed using the same standardized questionnaire set. If no response within 4 weeks of the mailed questionnaire or phone call then up to three reminder notifications were performed at two week intervals. Health status was serially assessed using validated, self-administered questionnaires for quality of life using the EuroQOL (EQ5D-5L). This is a widely used standardized instrument for measuring generic health status whereby higher scores represent better quality of life ( from −0.59 – 1.00 scale).(12) We recorded the illness perception using the validated brief illness perception questionnaire (B-IPQ),(13) anxiety and depression according to the PHQ–4(14) and treatment satisfaction (TSQM-9)(15). At six months, the patients’ anginal symptoms were assessed using the same standardized questionnaire set. If no response within 4 weeks of the mailed questionnaire or phone call then up to three reminder notifications were performed at two week intervals.

# Table 1 - Diagnosis and stratified treatment of coronary vasomotion disorders.

| ***Endotype*** | ***Diagnosis: Coronary vasomotion disorder*** | | ***Stratified medical therapy*** |
| --- | --- | --- | --- |
| **Microvascular angina** | IMR ≥ 25  (Microvascular resistance) | Index of microcirculatory resistance (IMR; abnormal ≥25 ). IMR is a quantitative method for specifically assessing microvascular function independent resting hemodynamics. IMR = distal coronary pressure * transit time (average time for x3 sequential saline bolus injections during hyperemia). | **Baseline therapy**: Consider aspirin, statin and ACE inhibitor therapy in all patients. PRN sublingual GTN  **Antianginal therapy**  **1^st^ Line – Beta blocker** (e.g. nebivolol 2.5mg OD or carvedilol 6.25mg BD uptitrated)  **2^nd^ Line - Calcium channel blockers (CCB)** substituted (Non DHP e.g. verapamil 40mg BD uptitrated) - where β-blockers are not tolerated or ineffective.  **3^rd^ Line – Add in therapy** (avoid long acting nitrates)  •CCB - DHP (e.g. amlodipine) – only for those on beta-blockers  •Nicorandil (5mg BD, uptitreated)  •Ranolazine (375mg BD, uptitrated)  Avoid long acting nitrate unless previously established good response or co-existent epicardial spasm |
|  | CFR < 2.0  (Coronary vasorelaxation) | Coronary flow reserve by thermodilution (CFR) <2.0 This reflects limited ability to increase coronary flow above the resting flow. |  |
|  | Microvascular spasm to Ach  (Propensity to microvascular constriction) | Angina during acetylcholine (ACh) infusion or bolus with typical ischemic ST-segment changes and epicardial coronary constriction <90% reduction in epicardial coronary artery diameter. Represents inappropriate susceptibility microvascular constriction. |  |
| **Vasospastic angina** | Epicardial spasm (>90%) | - >90% epicardial coronary diameter reduction with Ach (bolus up to 100mcg) compared with diameter after GTN. - Reproduction of angina symptoms - ST segment deviation on the ECG. | **Baseline therapy**: If atherosclerosis or endothelial impairment, aspirin, statin and ACE inhibitor should be considered. PRN sublingual GTN  **Antianginal Rx**  **1^st^ Line – Calcium channel blocker (CCB)** - e.g. verapamil 40mg BD uptitrated  **2^nd^ Line – Add Nitrate** - e.g. isosorbide mononitrate 10mg BD  **3^rd^ Line – Change Nitrate to nicorandil**- e.g. Nicorandil 5mg BD |
| **Non-cardiac** | Nil | FFR >0.80 with normal IDP (CFR >2, IMR <25 and negative Ach testing). | Cessation of antianginal therapy. Stop antiplatelet and statin unless other indication. Consider non cardiac investigation or referral where appropriate (e.g psychological referral, gastroenterology) |
